# The effect of acupoint therapy on sleep quality of hemodialysis patients: A protocol for systematic review and meta-analysis

**DOI:** 10.1097/MD.0000000000028182

**Published:** 2021-12-23

**Authors:** Gang Xiong, Lijun Hu, Chenglian Hu, Yunhua Yao

**Affiliations:** a Emergency Intensive Care Unit, The Central Hospital of Enshi Tujia and Miao Autonomous Prefecture, Enshi, Hubei Province, China; b Hospital Office, The Central Hospital of Enshi Tujia and Miao Autonomous Prefecture, Enshi, Hubei Province, China.

**Keywords:** acupoint therapy, hemodialysis, meta-analysis, protocol, sleep

## Abstract

**Background:**

Hemodialysis patients usually have sleep disturbance at varying degrees, which seriously affects the therapeutic efficacy and quality of life. Therefore, improving the sleep quality of hemodialysis patients is the key during treatment. Acupoint therapy can improve the sleep quality of patients. However, guidelines for improving sleep quality of hemodialysis patients by acupoint therapy are scant. This study aims to evaluate the effect of acupoint therapy on sleep quality in hemodialysis patients through a meta-analysis, providing clinical evidences.

**Methods:**

Randomized controlled trials (RCTs) reporting the effect of acupoint therapy on sleep quality in hemodialysis patients published before November 2021 will be searched in the China National Knowledge Infrastructure, Chinese Biomedical Literature Database, Wanfang database, the Chinese Scientific Journal Database, PubMed, Embase, The Cochrane Library, and Web of Science databases. Eligible literatures will be screened according to inclusion and exclusion criteria and assessed for quality using the Cochrane Risk of Bias Assessment Tool. Meta-analysis will be performed using Revman 5.4 software.

**Results:**

This study will evaluate the effect of acupoint therapy on sleep quality in hemodialysis patients using the Pittsburgh Sleep Quality Index (PSQI).

**Conclusion:**

This study will provide a reliable evidence-based basis for conducting acupoint therapy to improve sleep quality in hemodialysis patients.

## 1 Introduction

Hemodialysis is preferred to patients with end-stage renal disease.^[1]^ With the continuous improvement and advancement of hemodialysis technology, the survival time of patients has been greatly prolonged.^[2]^ However, most hemodialysis patients suffer sleep disturbances such as poor sleep quality, insomnia and easy awakening, very low sleep efficiency, difficulty in falling asleep again after waking up, and daytime sleepiness.^[3]^ Sleep quality of hemodialysis patients seriously affects the therapeutic efficacy and quality of life, and even the survival time in serious cases.^[4,5]^ Therefore, how to improve the sleep quality of hemodialysis patients is a current research hotspot.

At present, psychotherapy, pharmacotherapy, and exercise therapy are main methods to improve sleep quality in hemodialysis patients.^[6–10]^ The effect of psychotherapy on patients is individualized with a long-term intervention, which may cause a series of adverse events such as drug resistance and mental inhibition.^[11]^ Exercise therapy requires a high level of patient mobility and may not be applicable to some patients.^[12]^ Acupoint therapy is simple, convenient, and highly efficacy in improving sleep quality without adverse events.

Patients intervened with long-term hemodialysis can develop complicated symptoms such as restless leg syndrome, itchy skin, thirst, and depression,^[13,14]^ leading to sleep disturbances and declined sleep quality. Conventional methods are usually ineffective in improving sleep quality in these population. Acupoint therapy originates from the traditional Chinese medicine, which has a long heritage and unique advantages for the treatment of insomnia. A series of acupoint therapies such as acupressure and auricular pressure have been validated effective in improving sleep quality, reducing the time to fall asleep and prolonging the effective sleep duration.^[15–20]^ Besides, acupoint therapy can also prevent and cure diseases, which is very beneficial to the treatment of hemodialysis.

However, the effect of acupoint therapy on sleep quality of hemodialysis patients is unclear. This study aims to evaluate the effect of acupoint therapy on sleep quality of hemodialysis patients through meta-analysis, thus providing an evidence-based basis for clinical development of acupoint therapy.

## 2 Methods

### 2.1 Protocol register

This meta-analysis protocol is based on the Preferred Reporting Items for Systematic

Reviews and meta-analysis Protocols (PRISMA-P) statement guidelines. The protocol of the systematic review was registered on Open Science Framework, and the registration number is DOI 10.17605/OSF.IO/KJFTE.

### 2.2 Ethics

The data for our studies are extracted from published literatures and do not require patient recruitment or collection of personal information. Therefore, ethics committee approval is not required.

### 2.3 Inclusion and exclusion criteria

#### 2.3.1 Inclusion criteria

(1) Publicly available RCTs reporting the effect of acupoint therapy on sleep quality in hemodialysis patients.
(2) Patients receiving hemodialysis treatment with sleep disturbance are the study subjects.
(3) Conventional care is provided in both control group and observation group, and acupoint therapy, such as acupressure, auricular pressure beans, and acupressure is additionally performed in observation group.
(4) Outcome is evaluated by the Pittsburgh Sleep Quality Index (PSQI).

#### 2.3.2 Exclusion criteria

(1) Full text is not available.
(2) No available data or incomplete data.
(3) Duplicate published literatures.

### 2.4 Searching strategy

Relevant RCTs published before November 2021 will be searched in the PubMed, Embase, The Cochrane Library, Web of Science, China National Knowledge Infrastructure, Chinese Biomedical Literature Database, Wanfang database, and the Chinese Scientific Journal Database according to PICO principles, using MeSH terms combined with free words. References in the eligible literature will be manually reviewed to avoid missing data. The searching strategy in the PubMed is summarized in Table 1.

**Table 1:** PubMed search strategy.

Number	Search terms
#1	Renal Dialysis[MeSH]
#2	Dialysis, Extracorporeal[Title/Abstract]
#3	Dialysis, Renal[Title/Abstract]
#4	Extracorporeal Dialysis[Title/Abstract]
#5	Hemodialysis[Title/Abstract]
#6	Dialyses, Extracorporeal[Title/Abstract]
#7	Dialyses, Renal[Title/Abstract]
#8	Extracorporeal Dialyses[Title/Abstract]
#9	Hemodialyses[Title/Abstract]
#10	Renal Dialyses[Title/Abstract]
#11	or/1-10
#12	Acupuncture Points[MeSH]
#13	Acupoints[Title/Abstract]
#14	Acupoint[Title/Abstract]
#15	Acupuncture Point[Title/Abstract]
#16	Point, Acupuncture[Title/Abstract]
#17	Points, Acupuncture[Title/Abstract]
#18	Acupressure [Title/Abstract]
#19	Auricular point[Title/Abstract]
#20	Ear point[Title/Abstract]
#21	Ear Massage[Title/Abstract]
#22	Ear buried seeds[Title/Abstract]
#23	Ear hole planted seeds[Title/Abstract]
#24	Auricular point pressing with bean[Title/Abstract]
#25	Auricular pressure[Title/Abstract]
#26	Auricular point sticking[Title/Abstract]
#27	Auricular therapy[Title/Abstract]
#28	Auricular pressure beans[Title/Abstract]
#29	Acupressure[Title/Abstract]
#30	or/12-29
#31	Sleep[MeSH]
#32	Sleep, Slow-Wave[Title/Abstract]
#33	Sleep, Slow Wave[Title/Abstract]
#34	Slow-Wave Sleep[Title/Abstract]
#35	or/31-34
#36	Randomized Controlled Trial[MeSH]
#37	Random∗[Title/Abstract]
#38	Clinic trial [Title/Abstract]
#39	or/36-38
#40	#11 and #30 and #35 and #39

### 2.5 Data screening and extraction

**Figure 1:**
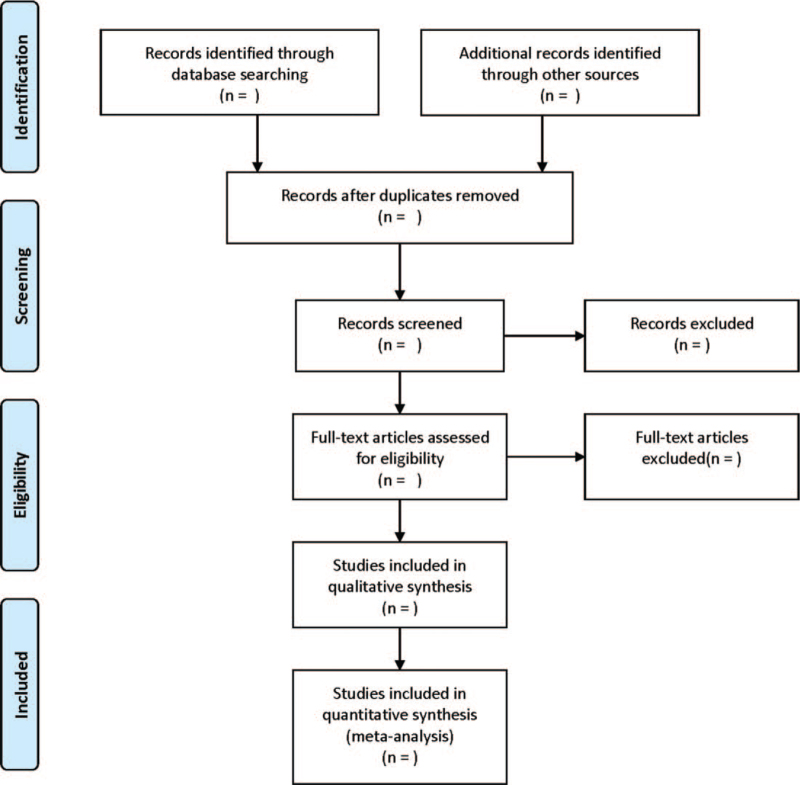
The PRISMA flow chart of selection process.

Two researchers will be independently responsible for the initial screening of retrieved literatures. After screening the titles and abstracts, the full text will be reviewed. Any disagreement will be solved by discussing with the third researcher. The following data will be extracted: first author and country, year of publication, sample size, age, interventions in control and observation groups, duration of intervention, and outcome indicators. Missing data will be requested by contacting the original authors through e-mail; Otherwise, it will be excluded. The searching process is shown in Figure 1.

### 2.6 Quality evaluation

The risk of bias assessment tool for RCTs recommended by the Cochrane Handbook 5.1.0 will be used to assess the quality of the included literature in the following aspects: randomization methods, allocation concealment, whether the investigators, subjects and outcome measures are blinded, completeness of outcome data, selective reporting of study results, and other biases.^[21]^ Each aspect will be evaluated into low risk of bias (satisfied), high risk of bias (not satisfied), or unclear risk of bias.

### 2.7 Statistical analysis

#### 2.7.1 Data analysis and processing

RevMan 5.4 will be used for statistical analysis. The standardized mean difference (SMD) and corresponding 95% confidence intervals (95% CIs) of the total PSQI scores will be calculated. Heterogeneity between included studies will be assessed by the Chi-square test. If *P* ≥ .1 and/or *I*^2^ < 50%, no heterogeneity is found between studies and a fixed-effects model will be adopted for combined analysis; Otherwise, a random-effects model will be used. Descriptive analyses will be performed if data cannot be combined.

#### 2.7.2 Subgroup analysis

Subgroup analyses will be performed according to the age, type of intervention, and duration of intervention.

#### 2.7.3 Sensitivity analysis

Sensitivity analysis will be carried out by one-by-one elimination method to test the stability of the combined effect values.

#### 2.7.4 Assessment of publication biases

Publication biases will be assessed by depicting funnel plots if more than 10 literatures are included.

## 3 Discussion

Hemodialysis is one of the effective methods to prolong the survival of patients with end-stage renal disease, but 45% to 80% of hemodialysis patients suffer from sleep disorders.^[2,22,23]^ It is reported that sleep disorders significantly affect the health-related quality of life and long-term survival of hemodialysis patients.^[24,25]^ Therefore, it is important to improve the sleep quality of hemodialysis patients, although conventional intervention methods are ineffective.

Acupoint therapy is guided by the theory of the External Treatment of Internal Diseases. Its therapeutic efficacy relies on the accurate selection of acupuncture points and the new technology of modern medicine. Acupoint therapy includes acupressure, auricular pressure bean, and acupressure massage.^[26,27]^ Acupoint therapy is able to stimulate the ear acupuncture points to accelerate sleep, prolong effective sleep duration, and improve sleep quality.^[28,29]^ A growing number of studies have shown that acupoint therapy is effective in improving the sleep quality of hemodialysis patients.^[27,30–33]^ However, its effect has not been systematically studied. There is no systematic and comprehensive meta-analysis on the effect of acupoint therapy on sleep quality in hemodialysis patients. This study aims to provide an objective evidence-based basis for the development of acupoint therapy.

However, our study also has some limitations.

(1) Most of the literature included in this study is published in Chinese language, which may result in publication bias.

(2) All included studies are performed in China, which has limitations in guiding other countries due to ethnic and cultural differences.

(3) The sample size is small, and more high-quality studies are needed for further analysis.

## Author contributions

Data curation: Lijun Hu.

Formal analysis: Chenglian Hu.

Methodology: Chenglian Hu.

Project administration: Yunhua Yao.

Supervision: Yunhua Yao.

Validation: Lijun Hu.

Visualization and software: Lijun Hu.

Writing – original draft: Gang Xiong and Yunhua Yao.

Writing – review & editing: Gang Xiong and Yunhua Yao.

**Conceptualization:** Yunhua Yao, Gang Xiong.

**Data curation:** Gang Xiong, Lijun Hu.

**Formal analysis:** Lijun Hu.

**Funding acquisition:** Yunhua Yao.

**Investigation:** Lijun Hu.

**Methodology:** Lijun Hu.

**Project administration:** Yunhua Yao.

**Resources:** Lijun Hu.

**Software:** Chenglian Hu.

**Supervision:** Yunhua Yao.

**Validation:** Chenglian Hu.

**Visualization:** Chenglian Hu.

**Writing – original draft:** Yunhua Yao, Gang Xiong.

**Writing – review & editing:** Yunhua Yao, Gang Xiong.
